# 2PNS++ point-cloud registration via hash of invariants and local compatibility check

**DOI:** 10.1371/journal.pone.0287134

**Published:** 2023-11-16

**Authors:** Hai Liu, Shulin Wang, Donghong Zhao

**Affiliations:** 1 Mechanical School of Jiangsu University, Zhenjiang, Jiangsu Province, China; 2 Yangzhou Polytechnic Institute, Yangzhou, Jiangsu Province, China; Universiti Teknologi Malaysia, MALAYSIA

## Abstract

In this research, we use hash match of invariants under fixed pair length and local compatibility check of positions or normal vectors to improve the efficiency of two-point normal set (2PNS) point cloud registration algorithm. On the one hand, we use the key value formed by the invariants of base point pairs of fixed length to construct and retrieve the hash table to realize the matching of base point pairs in the two point clouds to be registered to speed up the extraction of candidate transformation matrices. On the other hand, the time consumed in the verification phase is reduced by checking the compatibility between the positions or normal vectors of the corresponding points in the specific areas of the two point clouds under the transformation from the candidate matrix. Through these two improvements, the algorithm significantly reduces the time spent in the point cloud registration algorithm.

## Introduction

The development of 3D sensing technologies such as 3D scanner, range camera, and terrestrial laser scanner has made it more convenient to acquire 3D point clouds. 3D point clouds dare used in many fields such as reverse engineering, automatic pilot, control of robotics, protection of historical relics, and medical image etc. This situation puts higher demands on 3D point cloud processing technologies. As an indispensable step in point cloud processing, point cloud registration has received increasing attention. Point clouds obtained from different viewpoints need to be transformed into one complete point cloud. This process is called point cloud registration.

In detail, point cloud registration is a process of seeking the best rigid transformation T to align a point cloud P (source point cloud) with another point cloud Q (target point cloud), and expressed as T(P)≈Q under appropriate distance evaluation functions. According to whether the initial position alignment is required, point cloud registration usually be divided into two steps: coarse registration and fine registration. Coarse registration is also known as global registration because it can estimate the coarse transformation matrix without requiring the initial spatial position of the point cloud. However, the precision of coarse registration algorithms is often lower than fine registration algorithms. Fine registration is can obtain good registration results. In contrast, fine registration is also known as local registration, because without a good initial spatial location, it is likely to fall into the local optimal solution. As the most classical point cloud fine registration algorithms, the iterative closest point (ICP) method and its derivatives [1–6], have been widely employed due to their high efficiency and precision. In addition, to tackle outliers and noise, researchers raised point cloud registration methods with a probabilistic model, such as normal distribution transform (NDT) [7], coherent point drift (CPD) [8], hierarchical Gaussian mixtures (HGMR) and filterReg [9]. However, Whether the above mentioned algorithms can be successfully registered is closely related to the initial relative position between point clouds. Those algorithms belong to local registration algorithms and the approximate initial position of the point clouds from different views of a model to be registered is an indispensable requirement of those algorithms. To address this issue, several global registering techniques have been developed. An important technical and research focus is faster and more robust global registration algorithms of point clouds with acceptable accuracy.

The rest of this paper is organized as follows. In Section 2, related work is briefly reviewed. Section 3 describes the proposed algorithm detailedly. The experimental results on some datasets are shown and analysed in Section 4. Finally, there is the conclusion in Section 5.

## Related work

Most global registration algorithms for 3d point clouds use one of the following four ideas or their mixture: feature-based, random sample consensus (RANSAC)-based, machine learning-based and heuristic algorithms -based. The feature-based methods extract feature points and determine the corresponding relationship between point clouds from different views using feature descriptors that can remain invariant after a rigid-body transformation. For instance, according to this principle, registration is carried out, utilizing the Sample Consensus Initial Alignment (SAC-IA) algorithm based on fast point feature histogram (FPFH) [10], or similar algorithms based on signature of histograms of orientations (SHOT) [11] feature. When the feature is not evident, the noise is high, or the number of outliers is great, feature extraction becomes challenging, restricting registration speed or robustness. The original version of RANSAC-based methods relies on a brutal thorough search. These methods, such as the RANSAC algorithm, find the transformation matrix that makes the error function least by traversing the entire feasible transformation space. When compared to feature-based registration methods, these algorithms are more robust. However, when the number of points in a point cloud is large, these methods become unacceptably time-consuming. This is due to the fact that, when the overlapping rate is low, the RANSAC algorithm’s complexity is frequently its worst-case $O(N^{3})$ [12], where N denotes the point number of point clouds to be aligned. Later, the original RANSAC algorithm was improved and there appeared the four-point congruent set (4PCS) [13] algorithm, generalized 4PCS (G4PCS) algorithm, keypoint 4PCS (K4PCS) algorithm, Super4PCS (S4PCS) [12] algorithm, super generalized 4PCS (SG4PCS) algorithm, and 2PNS [14] algorithm. In addition, volumetric 4PCS (V4PCS) and adaptive 4PCS (A4PCS) have also appeared in recent years. Through the invariant features generated by machine learning technology [15–17], the machine learning-based method provides a relatively more robust transformation between two arbitrary point clouds. Typical representatives of such methods include point cloud registration network (PCRnet) [15] and deep global registration (DGR) [16]. Because the descriptors of machine-learning may contain more comprehensive features than geometric feature descriptors or other manual descriptors, feature extraction in point cloud registration can be more invariant. However, machine learning-based techniques require a lot of computing overhead during the training phase, and the algorithm’s interpretability is also subpar. Additionally, there are some registration methods that use heuristic algorithms. [18–20]. These global registration algorithms based on heuristic algorithms provide a additional idea, However, they typically result in a lot of computation time consumption.

Recently, a variety of fully automated global registration algorithms of the point clouds based RANSAC algorithms have played important roles in the point cloud processing field, which methods usually obey similar principles: With two partially overlapping point clouds as a given, repeated the verification process to compare pairs of congruent bases which were extracted from these two point clouds individually and try to find the optimal rigid transformation. Instead of the triplet used in RANSAC algorithms, the 4PCS algorithm developed by Aiger et al. uses the geometric information of congruent quadrilaterals on two models to calculate the transformation matrix between the two models. This improvement reduces the time complexity of the algorithm from $O(N^{3})$ to $O(N^{2})$. On basis of the 4PCS algorithm, the Mellado et al. introduced the S4PCS method, which is based on 4PCS, has an asymptotic complexity of O(N). The use of a grid-like data structure to look for point pairs within a particular distance range is the main advancement in the method.

In addition, Theiler et al. combined the 4PCS algorithm with keypoints and proposed K4PCS, which extracts keypoints from raw point clouds to be used as the input to 4PCS [21]. Mohamad et al. removed the planarity limitation of 4-points base set and introduced G4PCS [22] and its S4PCS-based version SG4PCS [23]. Raposo et al. [14] exploited normal vector information of point clouds and developed the 2PNS algorithm, which is a variant of S4PCS, aims at point clouds that are sufficiently dense and include many smooth regions and searches for correspondences between views using point pairs and the normal vector their endpoints rather than four coplanar points. Huang et al. [24] used the volumetric information of the points sets to make the extraction of the congruent bases faster and introduced V4PCS. Sun et al. introduced a more flexible congruent base extracting method and raised A4PCS, which is based on V4PCS and outperforms other variants of 4PCS in most cases [25]. Li et al. proposed an improved method call Super Edge 4PCS [26], which extracts boundary segmentation, overlapping regions of boundary segments and selects bases from them to register point clouds with obvious edges.

In this paper, we use two main improvements to reduce the time cost of the point cloud registration algorithm. The first is hash match of invariants under fixed base pair length of point clouds. The second is local compatibility check in the base neighborhood of point clouds. In addition, some improvements have been made in eliminating the ambiguity of normal vectors.

## Methodology

### Review of 2PNS

To clarify the proposed algorithm’s description, we first quickly review the 2PNS method so that the suggested algorithm’s explanation is obvious. The 2PNS algorithm was built on top of the S4PCS algorithm. The objective behind this approach is to make maximum use of point cloud normal vector information. This is done to estimate the right correspondence between two-point normal sets from which the rigid-body transformation can be computed, allowing the registration to be calculated robustly and cheaply. In detail, the 2PNS algorithm calculates five rigid-body transformation invariants {*d*, *θ*, *γ*_1_, *γ*_2_, *γ*_3_} of point pairs of both point cloud, finds corresponding pairs by matching these invariants and verifies these corresponding pairs to obtain the best match according the largest common point set (LCP). Here, d is the distance between two end points of a pair, *θ* is the angle between two normal vectors at two end points, *γ*_1_ is the angle of the first normal vector with the line segment connecting two end points, *γ*_2_ is the angle of the second normal vector with the line segment, and *γ*_3_ is the angle between the two vectors obtained by projecting two normal vectors onto the plane perpendicular to the line segment connecting two end points. This is not the same as the S4PCS algorithm’s quadrilateral search. This pair discovery reduces the number of possible search possibilities and, as a result, the amount of time it takes to complete the search. The essential steps of the 2PNS algorithm are depicted in follows:

Step 1: Randomly extract a pair of points from a target point cloud (P) as a long base pair (*A*,*B*) and calculate five rigid transformation invariants {*d*, *θ*, *γ*_1_, *γ*_2_, *γ*_3_}.
Step 2: Search for point pairs with the rigid transformation invariants of Step 1 in source point cloud (Q) as candidate pairs. The detailed search algorithm is similar to that in the 2PNS algorithm with linear complexity.
Step3: Compute the rigid transform matrix between the base pair in P and the candidate pairs in Q separately by singular value decomposition.
Step4: Apply the rigid transform matrix to original Q and calculate the LCP score between transformed Q and P.
Step 5: Repeat Step 3 to Step 4 until obtained congruent pairs are traversed.
Step 6: Repeat Step 1 to Step 5 until the termination conditions are met, such as given time is over, and then select the optimal transform matrix according LCP score.

The 2PNS algorithm computational complexity is half that of the S4PCS algorithm because it runs the pair extraction process over the target point cloud Q once. In addition, 2PNS algorithm does not need to extract congruent quadrangle after point pair extraction, which takes a large proportion of time to complete in the original S4PCS algorithm. The output of the experiment shows how the 2PNS algorithm is faster than the S4PCS algorithm for point clouds with enough density and smooth regions [14].

However, 2PNS still suffers from the problem of prolonged computing time in its verification stage, especially in the case of low overlap rate, when a large number of bases need to be tried to obtain the global optimal solution. To address this problem, we enhanced the original 2PNS algorithm by combining it with local compatibility of position and normal vector in this paper.

### Proposed algorithm

In this section, we describe an algorithm derived from the 2PNS algorithm with two improvements. The first is using base point pairs and congruent point pairs with a fixed length to significantly reduce time spent in extracting congruent point pairs. The second is using local compatibility of corresponding positions and normal vectors under candidate transformation matrices before the congruent point set verification stage to filter the fault transformation matrices.

#### Hash of invariants under fixed pair length

After selecting a base point pair in point cloud P and calculating its invariants, extract all point pairs whose invariants {*d*, *θ*, *γ*_1_, *γ*_2_, *γ*_3_}are equal to the base point pair within a certain error range from point cloud Q. Select a new base point pair in the point cloud P, and the previously extracted congruent point pair will be discarded and need to be extracted again. This results in a large amount of calculation time consumption. If these congruent base pairs can be reused, a lot of calculation time can be saved. To do this, you must ensure that the length of the base point pair selected in point cloud P is a fixed value. According to this idea, we can select an appropriate length *L* at the beginning, which should be included in the overlapping area of two point clouds, and extract all point pairs of this length from point cloud P and point cloud Q to obtain two sets of $S_{P}$ and $S_{Q}$.

$$\begin{array}{c} S_{P}=\left\{\left(P_{i},P_{j}\right)|∥P_{i}-P_{j}∥=L,P_{i}\in P,P_{j}\in P\right\} \end{array}$$ (1)

$$\begin{array}{c} S_{Q}=\left\{\left(Q_{i},Q_{j}\right)|∥Q_{i}-Q_{j}∥=L,Q_{i}\in Q,Q_{j}\in Q\right\} \end{array}$$ (2)

Next, we can match between $S_{P}$ and $S_{Q}$ and extract two point pairs with four invariants {*θ*, *γ*_1_, *γ*_2_, *γ*_3_} approximately equal. On this basis, we calculate the candidate transformation matrices according to these two corresponding point pairs and verify these matrices.

**Fig 1:**
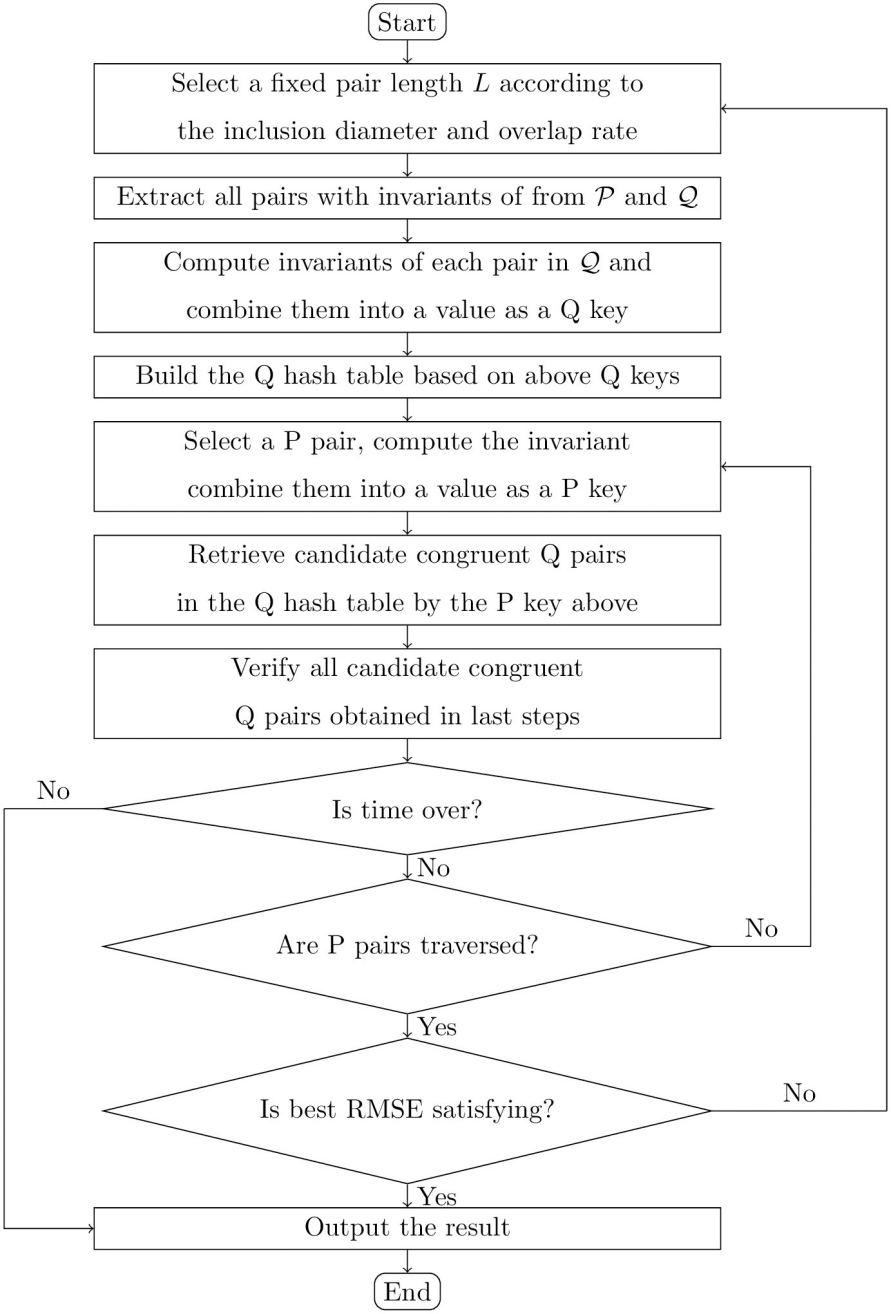
The flowchart of pair match between point cloud P and point cloud Q.

In the specific implementation, we use a hash table to complete the matching between $S_{P}$ and $S_{Q}$. More specifically, we use $S_{Q}$ to build a hash table. The index key value of the hash table is the value constructed by the four invariants of the point pair (*Q*_*i*_, *Q*_*j*_) in $S_{Q}$ as the key value. Accordingly, when we search the corresponding point pair in $S_{Q}$ for the point pair (*P*_*i*_, *P*_*j*_) in $S_{P}$, we also use the value constructed by the four invariants of (*P*_*i*_, *P*_*j*_) as the key value for retrieval. The flow chart of our algorithm is shown in Fig 1.

It should be specially noted that the selection of fixed point pair length *L*. Generally, *L* is selected according to the size and overlap rate of two point clouds. A simple way is to multiply the diameter of the point cloud by a specific proportion. Selecting the appropriate *L* means that the maximum point cloud spacing in the overlapping area is greater than *L*. In this case, it should be possible to extract the correct corresponding relationship of point pairs with a very high probability. If an extreme situation occurs and there is no approximately appropriate correspondence of pairs obtained after traversing all possible correspondences or the root mean square error (RMSE) obtained under the transformation matrix with the highest LCP score still does not meet the requirements, we should slightly reduce *L* and repeat the calculation process described above. Here, the equation of RMSE is

$$\begin{array}{c} RMSE=\frac{1}{N}\sum_{∥T\left(Q_{i}\right)-P_{j}∥\leq \delta }∥T\left(Q_{i}\right)-P_{j}∥ \end{array},$$ (3)

where *N* is the number of points of transform Q which exist corresponding points in P in the distance of *δ*.

#### Local compatibility of points and normals

Similar to the S4PCS algorithm, the verification step of the candidate transformation matrix of the 2PNS algorithm takes a large proportion of the calculation time. Reducing the time used in the verification step can effectively improve the efficiency of the 2PNS algorithm. In order to reduce the time consumed in the verification of obviously infeasible candidate transformation matrices, we need to eliminate these candidate transformation matrices first. It is easy to think of selecting several points on the point cloud to be matched to verify a candidate transformation matrix. However, there are two problems that must be faced. On the one hand, for two partially overlapping point clouds, the additional points selected for verification may not have corresponding points on the other point cloud. On the other hand, even for a matrix that is close to the best transformation matrix, it is difficult to determine the threshold value used to verify whether the positions of corresponding points coincide. For these two problems, we have imposed restrictions on the selection range of points for verification. Here we select the points within the neighborhood of the points in the corresponding two congruent point sets to verify these candidate transformation matrices. In general, it is not appropriate that the points used in the two congruent point sets used to calculate the candidate transformation matrix are isolated points. Therefore, under the approximate optimal transformation matrix, the probability of corresponding point pairs in the neighborhood of these points is significantly higher than that in other regions. In addition, the range of distance change between the corresponding points in these neighborhoods under the approximate optimal transformation matrix will be limited to a small range. Therefore, a more definite value can be taken to determine whether the positions of corresponding points coincide.

**Fig 2:**
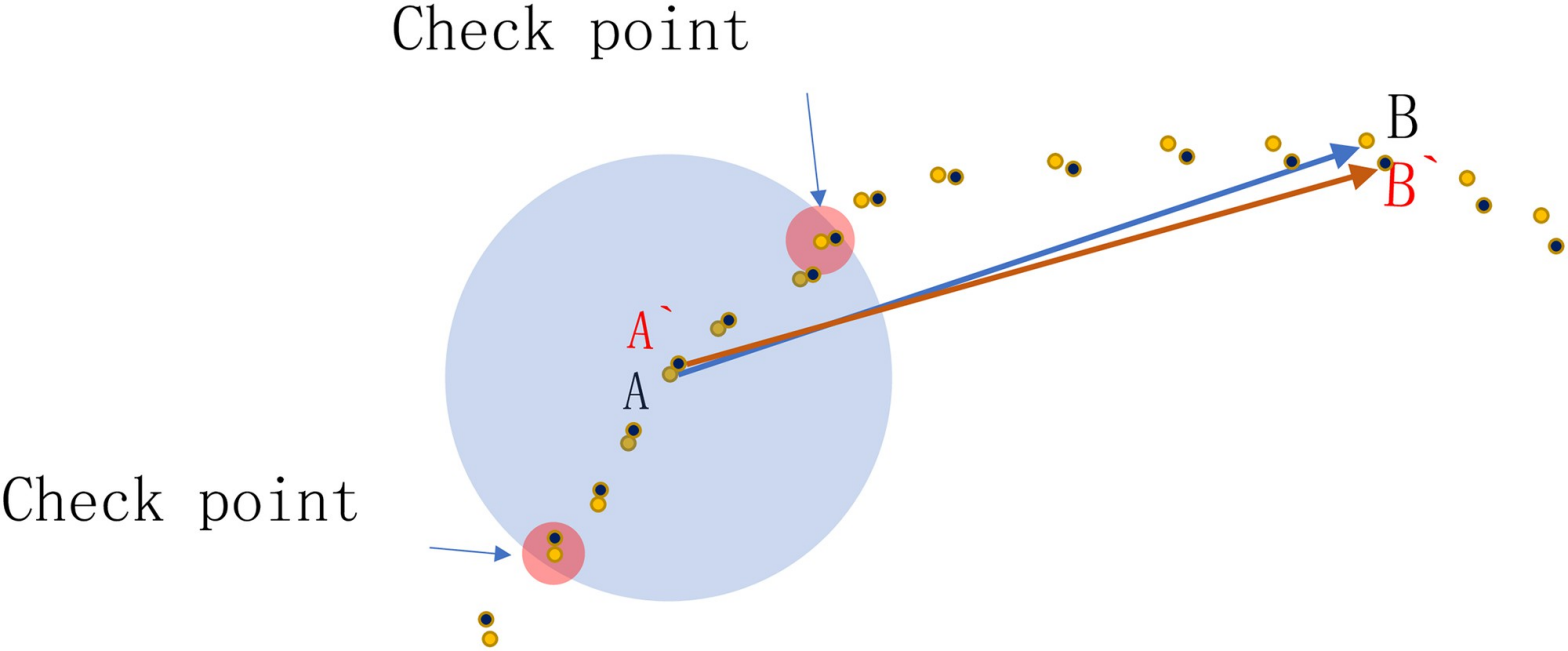
The illustration on the selection of check points in the neighborhood of base points(*AB* is a pair of points in P and *A*′*B*′ is in Q).

**Fig 3:**
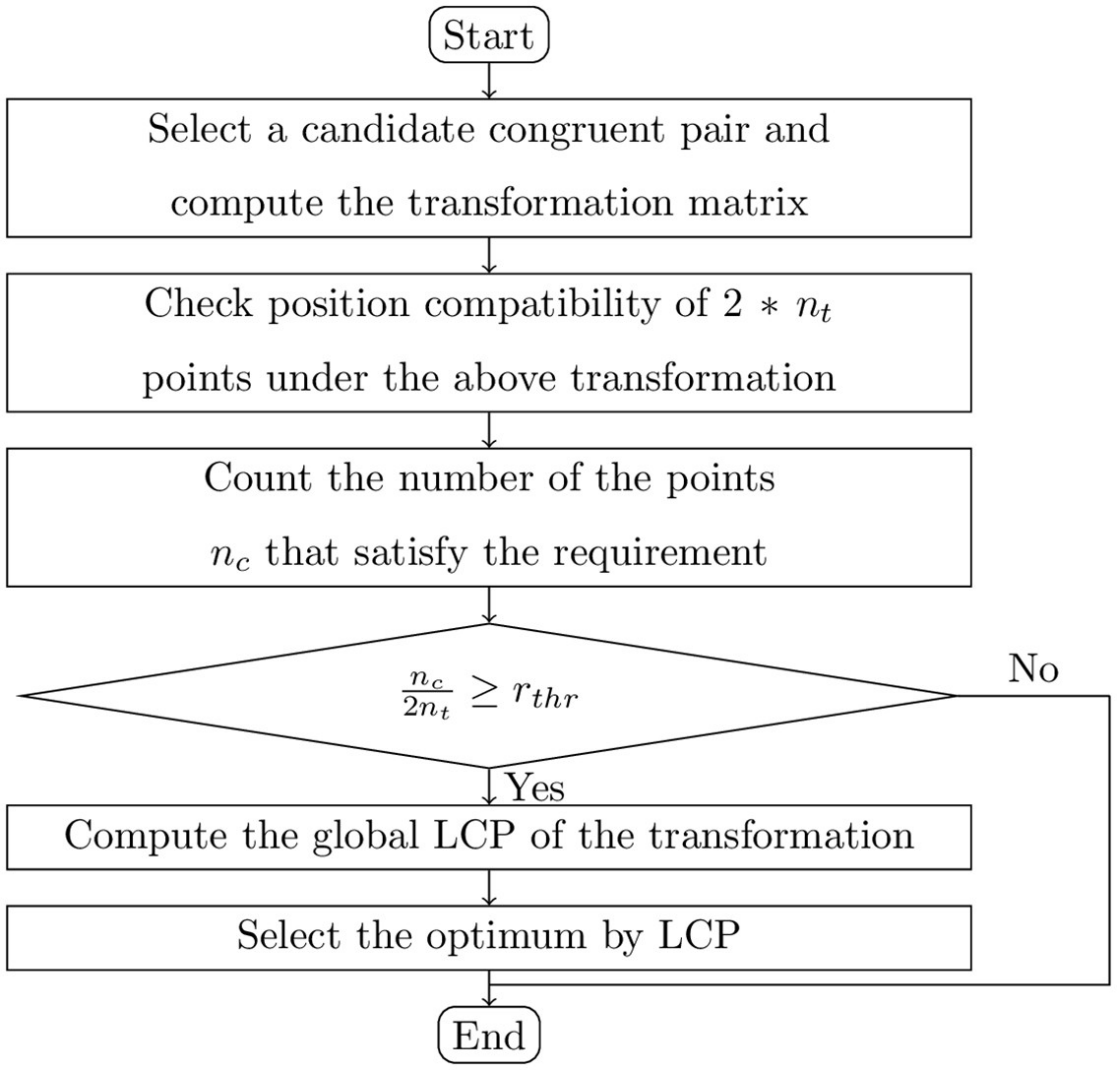
The flowchart of verification with local compatibility check.

To be detailed, there are a base point pair in P and its candidate congruent point pairs in Q when using the 2PNS registration algorithm. When we select base points in P, we check and exclude those isolated points and points near the boundary as base points. Here, we select *n*_*t*_ points in the neighborhood of each base point, which are farthest in the neighborhood from the base point and described as Fig 2. Then we use the method similar to the 2PNS algorithm to extract the point pairs that meet the requirements of five rigid transformation invariant parameters. Before every final LCP computation, we need to check whether the points extracted in the neighborhood of base points in P mentioned above have corresponding points in Q under the candidate transformation to verify whether the transformation matrix is roughly feasible. If

$$\begin{array}{c} \frac{n_{c}}{2n_{t}}\geq r_{thr} \end{array}$$ (4)

is satisfied, we can accept the candidate transformation matrix for the further LCP verification on all points, where *n*_*c*_ is the number of points in such *n*_*t*_ points that have corresponding points in the threshold distance *δ* and *r*_*thr*_ is the threshold ratio. If Eq (4) is not satisfied, the respective candidate transformation matrix will be rejected and the next correspondence verification will be started. The flowchart of the improved verification of candidate matrices is showed as Fig 3, which corresponds to the verification step in Fig 1.

For the point cloud with smooth surface, gentle normal vector change and high sampling density, we can also add the check of compatibility of normal vector between corresponding points to strengthen the screening of candidate transformation matrices. Although the additional filtering process will add additional time costs, these time costs are insignificant compared with the time spent in the original algorithm to calculate LCP at all points of the entire point cloud.

## Experiments and results

In this section, we conducted two experiments. The first experiment is about selecting the length L of point pairs. The second experiment is about comparing the effectiveness of the algorithm proposed in this article with other algorithms. The computational environment for the assessment is Intel i5-9600 at 4.6 GHz and 16 GB of RAM.

**Fig 4:**
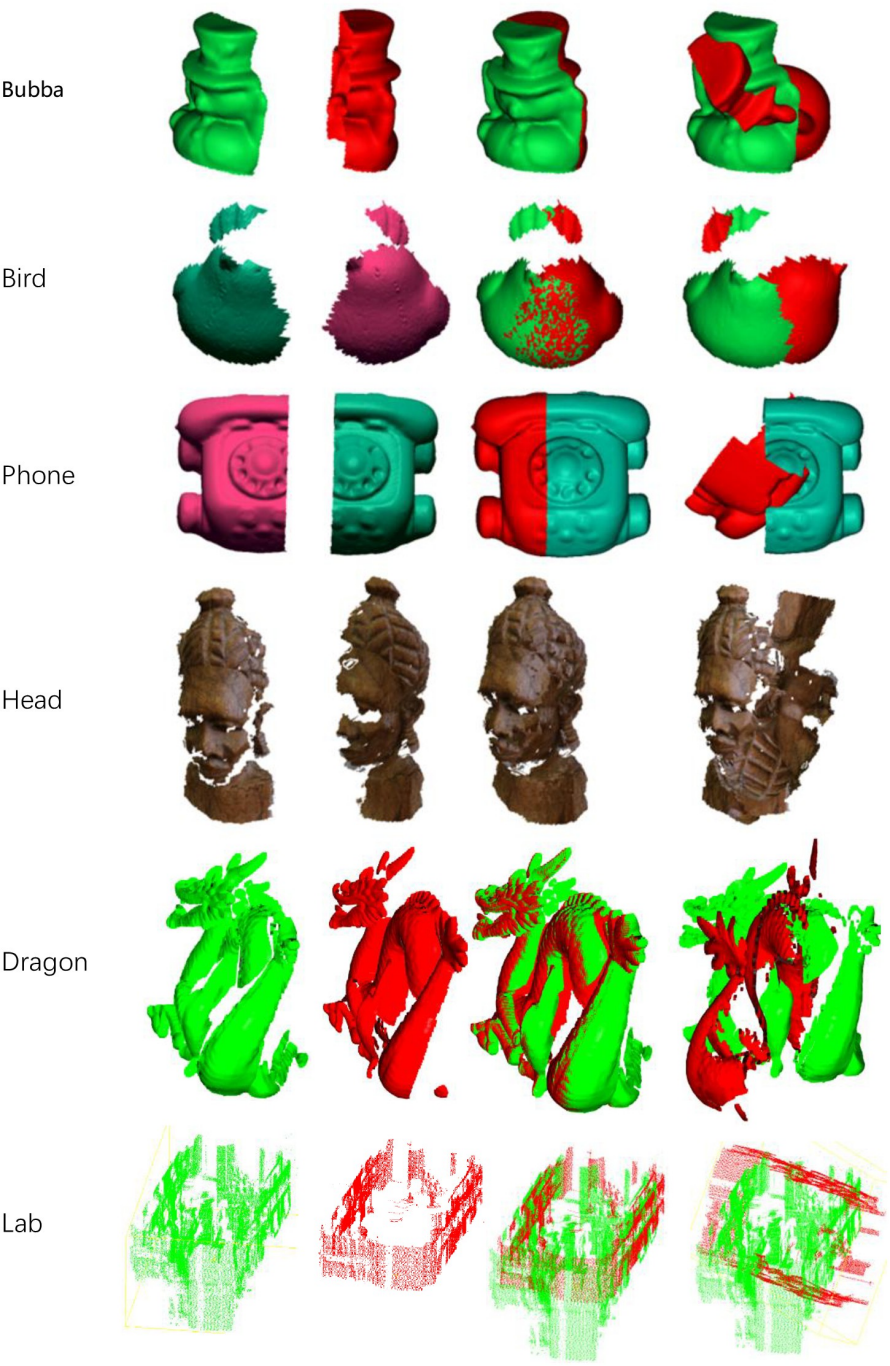
The illustration of models for the assessment.

Table 1 shows the relationship between the registration time and the ratio of L to the maximum radius of point clouds. Here we choose Dragon model, Phone model and Bubba model showed in Fig 4 for the experiment. The first row in the table contains the model names, followed by their respective overlap rates. The *Ratio* columns in the table denote ratio of L to the maximum radius and the The *Time* columns denote the average time spent using corresponding ratios for registration. The value *NaN* means that a valid result cannot be obtained here. From the table, we can find that the larger the overlap rate, the larger the upper limit value of *L*. In addition, within the range of *L* where valid results can be obtained, the larger *L*, the less time consumed. But if *L* exceeds the upper limit, the registration will fail. Based on experimental data, the initial value we set for the ratio here is 1.5 times the maximum radius. We used this expression to calculate the initial value of *L* in the second experiment.

**Table 1:** Relationship between spent time and the ratio of *L* to the maximum radius of point clouds.

Dragon 85%		Phone 55%		Bubba 24%	
Ratio	Time(s)	Ratio	Time(s)	Ratio	Time(s)
0.20	0.3	0.20	0.66	0.20	0.49
0.25	0.3	0.25	0.53	0.25	0.27
0.30	0.3	0.30	0.51	0.30	0.51
0.35	0.19	0.35	0.51	0.35	0.2
0.40	0.2	0.40	0.51	0.40	0.15
0.45	0.19	0.45	0.46	0.45	0.24
0.50	0.14	0.50	0.28	0.50	NaN
0.55	0.15	0.55	0.24	0.55	NaN
0.60	0.12	0.60	0.23	0.60	NaN
0.65	0.1	0.65	0.21	0.65	NaN
0.70	0.07	0.70	0.21	0.70	NaN
0.75	0.07	0.75	0.15	0.75	NaN
0.80	0.04	0.80	0.16	0.80	NaN
0.85	0.03	0.85	0.32	0.85	NaN
0.90	0.03	0.90	NaN	0.90	NaN
0.95	0.02	0.95	NaN	0.95	NaN
1.00	0.02	1.00	NaN	1.00	NaN
1.05	NaN	1.05	NaN	1.05	NaN
1.10	NaN	1.10	NaN	1.10	NaN

In the second experiment, we verified the efficiency of the proposed registration algorithm. The comparison algorithm here includes S4PCS and A4PCS on these point cloud models is in terms of accuracy and speed. The experiments were run on part of point cloud models used in the paper of S4PCS and A4PCS partly shown in the first two columns of Fig 4. These models are with different overlap ratio, levels of noise and sizes. In order to evaluate the registration processes quantitatively, one of different views of each model used in the evaluation is transformed with several random initial positions from the other. Part of transformed results are shown in the fourth column of Fig 4.

Here we used the metrics of quantitative assessment similar to those used in the paper of A4PCS. The first metric is efficiency, which is denoted by the average runtime of 30 runs. The second metric is accuracy, which is expressed in RMSE.

Table 2 shows the the comparison result between the proposed algorithm and the A4PCS algorithm. Here we use *T*_*C*_ = (*T*_*A*_ − *T*_*P*_)/*T*_*A*_ and *RMSE*_*C*_ = (*RMSE*_*A*_ − *RMSE*_*P*_)/*RMSE*_*A*_ to clearly demonstrate the effect. Two points can be seen from the table. On the one hand, for point clouds formed by point clouds with gentle changes in surface normal vectors, the registration effect is significantly improved, such as Bubba and Phone, where Bubba’s improvement effect is particularly significant. In addition, the Lab contains a large number of planes, which can significantly enhance the effect. In contrast, for the point cloud formed by the point cloud whose surface normal vector changes sharply, its registration effect is not obvious, such as HEAD. This is because the local compatibility check of candidate matrices is easy to make mistakes when registering point clouds with sharp changes in the normal vector of the surface. On the other hand, the registration efficiency of point clouds with more sampling points is also significantly improved [25], such as Lab. This is due to the large number of sample points generated by point clouds that need to be confirmed that there are more candidate matrices, and there is room for the advantages of local compatibility check.

**Table 2:** Comparison of registration efficiency and accuracy.

Models	Sample Size	A4PCS		Proposed		Comparison	
		*T*_*A*_(s)	*RMSE*_*A*_(mm)	*T*_*P*_(s)	*RMSE*_*P*_	*T*_*C*_(%)	*RMSE*_*C*_(%)
Bubba	200	1.25	3.56	0.33	1.16	73.24	65.34
Bird	200	1.12	3.01	0.43	2.71	61.53	11.00
Phone	200	1.02	5.09	0.32	3.72	68.26	31.15
Head	500	0.55	3.20	0.50	3.31	8.20	3.79
Hippo	200	0.71	3.40	0.34	3.25	52.13	5.56
Buddha	500	1.10	17.70	0.56	17.91	48.98	2.20
Dragon	500	1.23	0.46	0.54	0.42	56.25	11.11
Shelf	300	0.68	3.18	0.58	2.80	14.95	10.87
Cups	200	1.05	1.31	1.02	1.26	2.42	5.72
Tablel	400	1.40	14.59	0.73	14.62	47.73	7.78
Table2	600	2.71	28.19	2.06	25.84	24.26	8.33
Lab	1000	12.08	41.77	6.51	18.28	46.15	55.22

## Conclusion

Nowadays, the field of processing 3D point clouds uses registration frequently. Based on the 2PNS algorithm, the algorithm proposed in this paper accesses the hash table by encoding the invariants of fixed-length base point pairs to efficiently search for potential corresponding base point pairs. Furthermore, the algorithm also uses local compatibility check to filter candidate transformation matrices. The results of the experiments demonstrated that these improvements can significantly increase registration efficiency.

However, the proposed algorithm is not suitable for models with a lot of broken surfaces or where the surface curvatures of point clouds change dramatically, and the normal vector is abrupt. In the future, we plan to propose more suitable extraction of normal vectors and make the invariants of corresponding points more stable to expand the application scope of the proposed algorithm.
